# Farewell to Books

**Published:** 1872-02

**Authors:** 


### HALL'S

### JOURNAL OF HEALTH.

VOL. XIX.—FEBRUARY, 1872.—NO. II.

### FAREWELL TO BOOKS.

"WHEN Southey, at the end of life, fell into mental decay, he used to walk around his library, take book after book from the shelves, look at the binding and title, and, without ability to read, seemed to commune with them as with old friends on the edge of separation. I am becoming an old man now, and, looking at my books, the accumulations of some forty years, the melancholy feeling comes over me that we, before long, shall have to part."

Thus beautifully writes the author of "Among my Books," in the first of a series of essays, written originally for the "New York World," and subsequently demanded by the public to be put in book form, as too precious to perish with a daily newspaper.

Hale & Son, of New York, have done so well by the first edition, that a second was immediately called for, containing twenty-one chapters of sketches of Swift, Bolingbroke, Junius, Clarendon, and on other subjects; but the paragraph quoted will strike every scholar with peculiar force, the greater, as he is older. The old man loves his oldest books better than his newest, and at any moment of quiet leisure, he runs his eye along the shelves, and regards with a kind of affection the faded volumes of another age, scratched, and marked, and torn, and tattered.

Ask him what he would take for the most dilapidated one, and he will quickly say, " Money cannot purchase it." A dozen editions of it may have passed through the press since, each with its " valuable additions," but that old-time cover has peculiar attractions to him; he knows about what part of the book, and at what part of the page some sentiment, or fact, or incident is recorded, which impressed itself upon his memory, ever so many years ago. He looks at the volume again; he may have a consciousness that in all probability he may never have occasion to open it, yet there is a feeling that he might have to do so, and he instinctively exclaims, " Spare that book — don't move it from its place! " and he looks upon it again, as with the longing eyes of a friend. And many a time as he passes by in a hurry, the wish half expresses itself, " How I would delight to have the time to take each one of you down, and turn over your leaves, and think of the past! "

Some of these books, may be, were purchased in childhood. There is the old geography, and there, too, the little old-fashioned Latin grammar, with its quaint leather cover; and on its fly-leaf is your own name, in the handwriting of early childhood, when you had not reached a dozen years. But the teacher, where is he ? — the parents, who gave you the money to make the purchase; and those who stood by you in the class, with their full cheeks, and bright eyes, and curly locks, and mischievous mien ? Most of them dead and gone! Some are left to battle with the world; some occupy seats of honor, usefulness, and distinction. The majority, the great majority, have gone down into the grave; some on the battle-field, some by a suicide's hand; some have met the drunkard's fate. Others lived and labored for the best interests of their kind; but their works of love are ended; there was triumph in their death, and we who are left are only waiting to join in their immortal pæans

"WHENE'ER THE SUMMONS COMES."

But the brain need never wear out, like Southey's, if the health of the body is taken care of through life; taken care of, by learning early to live soberly, temperately, genially, busily; so far from wearing out, it works brighter, and clearer, and more acutely, till life's last hour. To be busy always, pleasurably busy, a little driven, not overmuch, this is the great secret of a healthy, happy life. It is the mistake of multitudes to look and labor for the time to come when they shall have nothing to do, that is, be compelled to do nothing; when they shall have a life of elegant leisure. No man was ever made to be a loafer, and all of us would do well to make a note of it, that until heaven is attained, entered, our true work is never done.

